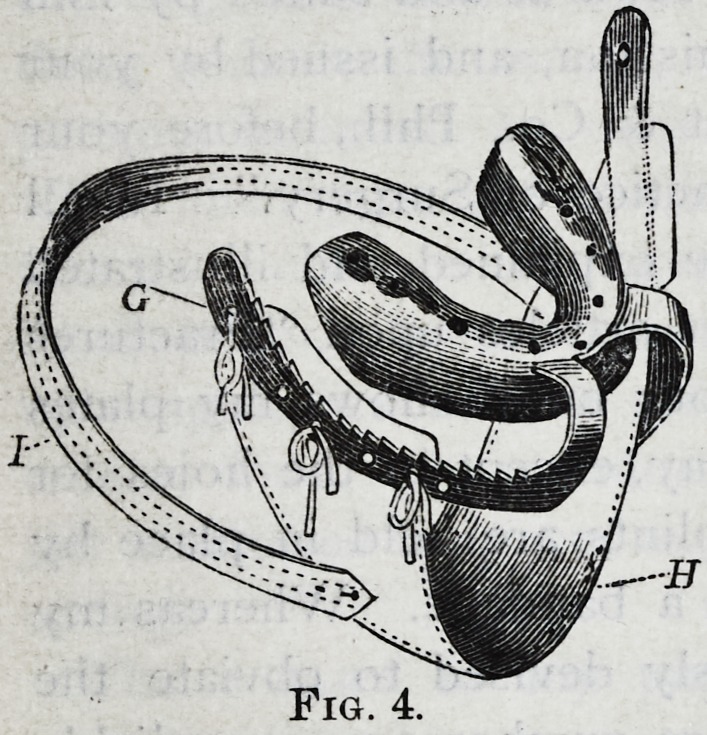# Treatment of Fracture of the Jaw, with Critical Remarks, as Sent to Prof. D. Hayes Agnew, M. D.

**Published:** 1884-01

**Authors:** Thomas Brian Gunning

**Affiliations:** New York.


ARTICLE II.

### TREATMENT OF FRACTURE OF THE JAW, WITH CRITICAL REMARKS, AS SENT TO PROFESSOR D. HAYES AGNEW, M. D.

BY THOMAS BRIAN GUNNING, D. D. S., NEW YORK.

The four splints peculiar to my treatment, illustrated by cuts and also by selected cases in which they had been used together with full explanations as to their manufacture and application were published in the *New York Medical Journal* for September and October 1866; The *British Journal of Dental Science* also of 1866; *Dental Cosmos*, Vol. VIII; AMERICAN JOURNAL OF DENTAL SCIENCE, Third Series, Vol. 2; and a synopsis is given in "Heath's Injuries and Diseases of the Jaws." Diagnosis of fracture of the jaws was not however dwelt upon; but as preparatory to this in 1867, I published my views of the muscular action which controls the lower jaw. (See *New York Medical Journal*, Vol. VI, p. 193; AMERICAN JOURNAL OF DENTAL SCIENCE. Third Series, Vol. 1, p. 597; *Dental Register*, Vol. XXII, p. 103.

Early in 1880 circumstances made it necessary that the subject should be again taken up and in the April number of the *Independent Practitioner*, I commenced a series of articles which after showing the action of the muscles involved and speaking at length upon the diagnosis of fractures of the lower maxilla, the closing paper again gave a clear view of the four splints used in my treatment of these injuries.

These repeated presentations of the splints have however proved insufficient to correct the misrepresentations which have appeared to confuse and mislead the reader; and the three years which have just closed, show a condition of affairs which calls upon me to take decided action in the matter; I therefore again bring forward the splints that the reader may judge as to my strictures on those who have so grossly misrepresented my treatment.

INTERDENTAL SPLINTS.

In the year 1840, when treating the first fractured lower jaw placed in my care, I found treatment by bandages unreliable. For, while the muscles tend to displace the bone, bandages frequently increase the difficulty; especially when swelling sets in through their pressure. They also, by interfering with the circulation, tend to prevent union. Teeth, loosened by the injury, are left unsupported, and the motions of the jaw, cheeks and lips painfully restricted.

Of the contrivances invented to supplement bandages, many were even more objectionable, and little improvement has been made in general treatment up to the present time. Having successfully used interdental splints, in many cases which have proved unmanageable under the usual treatment, I am convinced that they are superior to all other appliances.

When a well adapted splint is on the teeth and gum the other parts around the bone are, to a great extent, a counter support to the splint. Thus the broken jaw, together with any teeth loosened by the injury, is held securely in place, until the fractured bone is re-united and the teeth become firm. Meanwhile the motions of the jaw are in most cases unrestricted and the cheeks and lips always left free.

On February 12th, 1861, I applied a "vulcanized hard rubber splint" to the fractured jaw of a seaman in the United States Naval Hospital, and from the vulcanite splints used by me shortly after, I selected three which show all that is essential to hold any fractured lower jaw in place.

The fourth, a metal splint, is sufficient for the treatment of most cases, and can be applied by surgeons and country practitioners, who can also treat most cases of fracture with rubber splints, if assisted by the neighboring dentist.*

*The splints were described in a paper read before the New York Academy of Medicine, June 1st, 1864. For report of this, and the earlier presentation of the subject, see the *Academy's Bulletin*, Vol. II, pp. 82, 83, 84, 85, 153, 168, and 307, also "Transactions of the Medical Society of the State of New York, for February 1863:" *American Medical Times*, August 8th, 1863; *Dental Cosmos*, September, 1863. Handbuch

The radical and distinctive feature of these splints is, that, when suitable teeth are in the mouth, nothing is required on the outside, and the patient may move about. In the use of these splints fractures of the lower jaw are divided into two distinct classes; first, those in which the teeth and gum of the fractured jaw are alone used to control the fractured bone, and the jaw is allowed to move naturally; second, those in which the splint is fitted to both the upper and lower teeth, the jaw being held still; but no bandage is used around the head.

To apply these splints the fractured jaw should, if possible, be set and held by ligatures around the teeth while an impression of the teeth and gum is taken in pure warm wax confined in a cup like No. 4 splint; the plaster cast from the impression will then be precisely what is required to mould the splint. If the bone cannot be held in place an impression may be taken of the teeth in the best attainable position, the plaster cast then separated where necessary and the parts set in place; a cast of the upper teeth will guide in putting these parts of the lower cast in place.

FIG. 1.

The holes marked A go through the top of the splint for the purpose of syringing the part within with warm water during treatment. The dark round spots in all the cuts represent holes for similar purposes

FIG. 1 represents the inner surface of a splint which incloses all the teeth and part of the gum of the lower jaw, and merely rests against the upper teeth when the jaws are closed. This splint is adapted to the treatment of all cases which have teeth in both fragments.

*The angles of the jaw tend outward, when the jaw is fractured through the front.* It is therefore necessary that the splint should go down and extend back as far on the outside as

---

der Lehre Von Den Knochenbruchen von, Dr. E. Gurlt, Professor den Chirurgie an der Konighlichen Universitat zu Berlin, p. 438.

All these works give verbatim reports from the proceedings of the Academy of Medicine, January 7th, 1863.

the muscles admit, especially on the short fragment, if there is much difference between them. The parts near the external oblique line are so formed that the splint can be fitted to them perfectly, and the outer ends of the splint, should be quite thick, that they may be well rounded.

I have generally used this splint without any fastenings, but in children and even adults it is sometimes advisable to secure it by pack-thread or wire, or by screws passing into or between the teeth, or by the wings and band of Fig. 4.

When screws are used to hold any rubber splint fast on the teeth, metal nuts must be imbedded in the splint, for the screws to work in.

Small openings should be made opposite particular teeth, to observe how the jaw stands in the splint. This is important in all splints.

FIG. 2.

FIG. 2 shows a splint for cases in which it is found impracticable to hold the fragments together, except by keeping the fractured bone still; this splint, in addition to fitting the teeth and gum of the lower jaw, must also inclose the upper teeth, as shown in the cut, where screws may be seen opposite both lower and upper teeth.

By this arrangement the fragments of the lower jaw are secured not only relatively to each other, but also to the upper jaw.

This splint is therefore adapted to the treatment of *all fractures back of the teeth*, whether in the body, the rami, or their terminations. In these cases the splint may be cut away in front, and extended across the roof of the mouth, when there are upper and lower back teeth to fasten to, and thus give as much room as possible to speak and eat through. Opening the teeth a quarter or three-eights of an inch would not have any bad effect on the position of the fragments, even if the jaw were broken through the necks of both condyles, as the parts near the fractures would move but little and the back of the jaw could be raised high enough to keep the broken surfaces in contact. Even if the neck of one side only were broken, the lower part could be kept firmly up against the fragment above.

When the jaw is held fast to the upper teeth, especially when wings project between the lips, passages should be cut through the sides of the splint, where the absence of teeth or separation of the jaws gives a chance for the saliva from the parotid glands to enter the mouth, otherwise it may overflow at the lips.

FIG. 3.

F, upper wing. G, lower wing. H, mental band to hold the jaw up in the splint. I, neck strap to keep the band back K, balance strap to hold the cap in place.

Fig. 3 shows the wings for cases having no teeth in either jaw—the ends of the wings within the mouth being imbedded in a vulcanite splint similar in principle to that of Fig. 2.

Wings made of steel or iron may be quite light. They should have small holes every half inch to hold the strings, lacing, etc. The arch of the wings should be high enough to give the lower lip room to go well up. The wings for each side of the jaw are in one piece, and the parts within the mouth pass back in the line of the upper gum. They are thinned down and pierced with holes, that the rubber in which they are imbedded may hold them firmly.

The tape strings pass from the cap inside and under the upper wings, then up between them and the tape lacings, which keep the strings from slipping, to the cap whence they started. The mental band (which is only one thickness of linen,) passes up between the sides of the lower jaw and the wings where it is tied by the strings, which pass through the holes. The band is cut off to show this; but when worn it should be turned down on the outside and pinned just below the wings. The neck strap should be sewed to the mental band on one side and pinned on the other, and worn tight enough to keep the band from slipping forward over the chin.

The jaw and splint are supported by the cap forward of its centre. This is counterbalanced by the elastic strap which passes from the back of the cap down around an unelastic and much heavier strap, extending across and fastened to the shoulders by elastic ends. The balance strap returns to the cap and is buckled tight enough to hold the jaw up. At night it may be slackened to do this, with the neck flexed. It slides on the shoulder strap as the head inclines to either side.

By this arrangement the splint is a resting place for the broken jaw, while the wings give firm attachment to appliances which hold the jaw up with the least possible pressure upon the external parts, as the wings need not press either against the jaw or the zygomas.

FIG 4.

G, wing of malleable iron, projecting with its fellow, from the splint to which they are soldered. H, mental or splint band, with the end left up to show the manner of tying it. I, neck strap. The mental band is made of linen or any thin material.

Fig. 4 represents a splint devised in 1863, for the use of practitioners out of the reach of a dentist, and for hospital use. This splint is made of cast tin, and is applied with a lining of gutta-percha. It is in the shape of an impression cup, and seven sizes are kept ready for use from which one can be selected for the broken jaw. The wings are of malleable iron, tinned to prevent rusting and for more readily soldering. Three sizes are sufficient to select from.

The splint has a handle in front, that it may be used as a cup to take the impression of the jaw—the holes being used to allow a small probe to be pressed through the wax, down to the teeth, thus allowing air to enter to facilitate the removal of the impression, and when in use as a splint giving entrance to warm water thrown from a syringe, to keep the parts clean.

The splint should be made to fit well by bending, cutting off the edges and rounding them up smooth. When a tooth projects so as to keep the splint from fitting, a hole may be cut to let the tooth through, if the metal cannot be hammered out. This should all be done before taking the impression, as a well fitted cup assists greatly in this important matter.

After the *cast* is obtained, the handle in front should be cut off, and the wings, *if needed*, soldered on, care being taken that their edges are clear of the corners of the mouth when *open*. Warm gutta-percha should then be placed in the splint, pressed down on the cast, and, after cooling in water, the softened plaster should be dug out

This splint has the advantage of being easier of application, and can be applied in much shorter time than a rubber splint, especially if the fractured bone can be set and held by ligatures firmly enough to bear the pressure of the warm gutta-percha for the splint can then be at once applied to the teeth, and the gutta-percha closing around them, the bone will be kept in place without other fastenings.

When the fragments of the jaw cannot be held firmly enough to bear the pressure of warm gutta-percha without displacement, Plaster of Paris would hold the jaw securely in the splint for a long time. In these methods the ligatures are left on.

---

To D. Hayes Agnew, Esq., M. D. L. L. D., Professor of Surgery in the Medical Department of the University of Pennsylvania.

*Sir*:—In the preface to your recently completed work "The Principles and Practice of Surgery" you say: "In the composition of its pages, while I have expressed my own views independently on all subjects, I have also endeavored, as far as was consistent with the scope and limits of the work, to record those of other writers, not only that the student and the practitioner may be made familiar with the literature of their profession, but also that they may be able in their observation and practice to contrast different plans of treatment, and in this way draw their own conclusions in regard to the relative merits of the various modes of managing surgical disease. Whatever may be the defects of the work,—and none can be more sensible of these than myself,—I have endeavored most conscientiously to furnish a safe and reliable guide for the surgical practitioner."

FIG. 642.

Gunning's Interdental Splint. A points to perforations for injecting water.

FIG. 643.

Gunning's Interdental Splint.

With this in view, those for whose instruction you wrote could not suspect that the work contains statements which are untrue, and mislead in regard to the treatment of any important injury. Yet the section on "Fracture of the Inferior Maxillary Bone" contains such statements. To give a clear understanding of the matter to you, and to all who may read this letter, I quote from your article verbatim and remark upon the misrepresentations. In Vol. I, page 846 you refer to the interdental splints devised by me and used in treating fractures of the maxilla, as follows:

"Among the simplest of Gunning's splints are the forms shown in Figs. 642 and 643, which receive all the teeth of the lower jaw, extend a short distance over the gum, and have perforations through which to throw a stream of liquid for the purpose of cleanliness. This splint when placed in position forms a cap, and is kept in place by securing the jaws together with a bandage, or by means of screws passed between the teeth."

Now my splint No. 1. your figure 642 was expressly devised to be used without a bandage; it holds the fragments of the jaw in place by means of the teeth without anything external to the mouth, and it allows the jaw to move and to be used in eating and speaking; and this form of splint is *adapted* to the large proportion of fractures of the maxilla. If the patient can be depended on, never, however, if a child, this splint may in many cases only be fitted to the teeth, and without screws in or between the teeth, or any ligatures, the fragments of the jaw will be held firmly together.

For in eating or in closing the splint against the upper teeth the muscles carry the broken jaw up and keep the fragments in place; the muscles and the surrounding soft-parts forming a counter support to the interdental splint.

This splint No. 1, was first applied on Feb. 12, 1861. It was used on the jaw of a Spanish seaman in the Naval Hospital, New York, and it cured the patient, although he had been subjected to four months unsuccessful effort of the government surgeons, assisted by others in the vicinity. Thus the surgeons were spared the mortification of sending the man home uncured. A similiar splint was shown to the New York Academy of Medicine, Jan. 7, 1863 with another case in which it was used, then published with illustrations in their Bulletin; and in February brought before the Medical Society of the State of New York, as shown in the *Transactions for 1863*, and in the Medical Report of the Centennial Commission 1876 this splint was admitted to be the first splint ever used without an appliance outside the mouth. Surely this splint should have been fairly reported and truly described in your work on "The Principles and Practice of Surgery." Had this been done other sufferers could have the use of it; whereas your book misleads the surgeon and student in regard to it.

Even in the few injuries where the fractures are such that it is necessary to use the upper teeth as a base to hold the broken lower jaw still; as in fractures in the ascending ramus, or say all fractures back of the teeth, my splint No. 2, now shown, is not kept in place as you say by securing the jaws together with a bandage. This splint, like No, 1, holds the fragments of the jaw by means of the teeth only; without any bandage; and while the patient wears this splint they may follow as with No. 1, their usual occupations.

GUNNING'S INTERDENTAL SPLINT.

FIG. 644.

Of my splint No. 3 you say: "A third splint of Dr. Gunning's; one which he uses in cases where the teeth have been lost is formed by connecting steel branches with the interdental part of the apparatus, of which the upper branch passes along the superior part of the face, and the lower one along the outside of the lower jaw; these are kept in place by three bands, one being placed at the chin in order to hold the jaw up in the splint, one running from the metal band to the back of the neck, and one passing to a cap which is worn over the head, and with which the splint is connected."

This is my plate 3 with its reference letters cut away and your description leads the reader to suppose that a band of *metal* goes under the chin to hold the jaw up in the splint , and that *metal* bands are used instead of strings of tape to hold the splint by means of the cap and to keep a *metal* band from slipping over the chin. But no *metal* band is used any where nor spoken of by me. In the absence of teeth the wings are used, two on each side, the upper range over the malar bones and the lower along the jaw; and from the cap on the head tape strings pass down on each side to the upper wings and hold the splint against the upper gum, while the broken lower jaw is held up in the splint by a single thickness of linen or other thin material which extends across under the chin from one lower wing to the other; while the lips, cheeks and all the face are left free from presure.

This statement would place the splint plainly before your readers, and give them the use of it, for their patients, when they needed or preferred it. This description is also briefer than your deceptive text. Certainly this splint No. 3. (your figure 644,) deserves fair notice, it having been successfully used on the bad fractures of the Hon. William H. Seward subsequent to the attempt to assassinate him.

Surgeon General Barnes and Surgeon Basil Norris of the army, together with Dr. Whelan, chief of the Medical Bureau of the Navy, and others had signally failed to secure by ligatures and bandages the fractures received in falling from his carriage, before the Secretary was cut so terribly on the night that President Lincoln was killed.

Further I did not take charge of the case nor set the fractures until twenty five (25) days after the accident, fifteen after the attempt to kill him, yet this splint with upper wings held the double and compound fractures of the jaw securely for sixty-eight days without a moment's intermission.

I described this splint No. 3, to the New York Academy of Medicine, June 1, 1864, but the upper wings were never used until I applied them May 2, 1865, in Mr Seward's case.

Since then a severe fracture without a tooth in the mouth has been treated successfully; in which both upper and also the lower wings were used. It was applied in May 1879, to the jaw of a farmer 70 years old with such good results, by Dr. J. Adams Bishop; reported in Johnston's *Dental Miscellany* Vol. VII, p. 63 and in the *Independent Practitioner* Vol. II, p. 108. Thus the splint No. 3 has been fully tested, for this patient's fracture could not be held by the bandages used by the Physicians who first attended the case. I devised this splint for fractures without teeth to hold by, and it has proved to be a perfect control for such cases; for the Secretary of State attended to the duties of his office while wearing it, and the farmer walked around at once; and followed his plough and did heavy work before his splint was left off, although he wore it only six weeks.

The deception of your text in regard to my treatment of these injuries is made complete by leaving out my splint No. 4 here shown.

FIG. 4.

This splint made of tin and applied to the teeth of the fractured jaw by means of a lining of gutta percha, or of Plaster of Paris, was devised in 1863, for hospital use and for practitioners out of the reach of a dentist. It is cast with a handle in front, so that it is an impression cup such as dentists use, but when applied as a splint, the handle is cut off, and, if needed, wings are soldered on, and from these when the splint is worn a single thickness of roller passes under the jaw from one wing to the other.

I reported this splint to the New York Academy of Medicine June 1, 1864, in answer to their request and letter of thanks in 1863. Within the week after reading the paper I applied this splint No. 4 to the jaw of a boy under, Dr. Freeman's treatment and in July I used the *same* splint with a new lining, on the jaw of a boy sent to me by Dr. King. The indentations shown in the cut represent those made by the upper teeth of both boys when eating.

The splints Nos. 1, 2, 3, and 4, with cases to explain and illustrate the treatment are clearly shown in my paper on the "Treatment of Fractures of the Lower Jaw by Interdental Splints;" first published in 1866; they are also distinctly and fairly shown in every edition of that unique work "INJURIES AND DISEASES OF THE JAWS," BY CHRISTOPHER HEATH, F. R. C. S. published by John Churchill & Son, London, and by Lindsay & Blackiston, Philadelphia, (this work is the Jacksonian Prize Essay of the Royal College of Surgeons of England, for 1867.)

The splints are also described at length and favorably noticed in the Report of the Judges of Group XXIV on Medicine, Surgery and Prothesis, transmitted by the Secretary, J. H. Thompson, A. M., M. D. to Prof. Francis A. Walker, Chief of the Bureau of Awards and edited by him for the U. S. Centennial Commission, and issued by your own Publishers, J. B. Lippincott & Co., Phil., before your work, "The Principles and Practice of Surgery." In all these publications the splints are explained and illustrated by the same plates used in your articles upon "Fractures of the lower Maxilla." But your book shows my plates with the reference letters cut away, except to the holes for syringing, and states that the splints are held in place by securing the jaws together with a bandage. Whereas my interdental splints were expressly devised to obviate the use of these bandages which are cumbersome, unreliable and often destructive. These splints are not as you intimate merely supplemental; each one is a complete and reliable support. The first is for all injuries in which the fractured jaw is allowed in my methods to move naturally while under treatment and by far the larger proportion of fractures can be thus treated.

The second splint is for fractures in which the broken jaw is held in fixed relation to the upper one; and in some of these cases this splint does not cover the front teeth; so that when worn it is unseen.

All the splints have small openings to allow observation of the teeth which are near the fractures so that the position of the broken ends of the bone can be learned at any time without removing the splint; and in fractures in which the lower jaw is held in fixed relation to the upper one, the splint has channels for the saliva from the parotid glands to pass in around the tongue.

You leave these important devices unnoticed and cut away the letters of reference, yet in the text given (say 7 lines) to my splints 1 and 2, you twice remark upon keeping the splint clean, and twice say or suggest that they cover all the teeth of the lower jaw; and then leave your readers ignorant and misled in respect to the radical features of the splints. But in less than the room given to the repetition you could have told that these splints hold the fragments of the bone in place securely without any thing outside the mouth, are quite comfortable, and the patients attended to their business and move about as when their jaws are sound. They do this even when the fractures are so severe that the jaw is held in fixed relation to the upper teeth, for in such cases the opening in front affords room through which to speak and receive food. But in most fractures as before stated, the jaw is allowed to move; and the top of the splint is used in eating.

The 8 cases in my paper which show the complete control attained by means of these splints were carefully selected, and with the 4 cuts spoken of in this letter, place my treatment, shown fully in 1866 at the service of all. Mr. Christopher Heath quotes from the *New York Medical Journal* and the *British Journal of Dental Science* 1866, and his book shows my treatment clearly. In its appendix Case VI is my report verbatim of the Hon. Wm. H. Seward's case.

The Official Report of the United State Centennial Commission closes in respect to my treatment of fractures of the maxilla as follows.

"In connection with the splints shown, was a series of casts illustrating the double compound fracture of the jaw of the late Hon. Wm. H. Seward, showing the jaw broken on both sides between the bicuspid teeth. Also a double cast of the upper and lower jaw as held by the splint for sixty-eight days. As no teeth were left in the upper jaw, the wings and cap were used as shown in Fig. 3. The result was thoroughly satisfactory.

The Secretary Dr. J. Henry Thompson who transmitted this report of the Judge of Group X X I V was a resident of Washington. D. C. where I treated the Secretary of State.

[TO BE CONTINUED.]

## Figures and Tables

**FIG. 1. f1:**
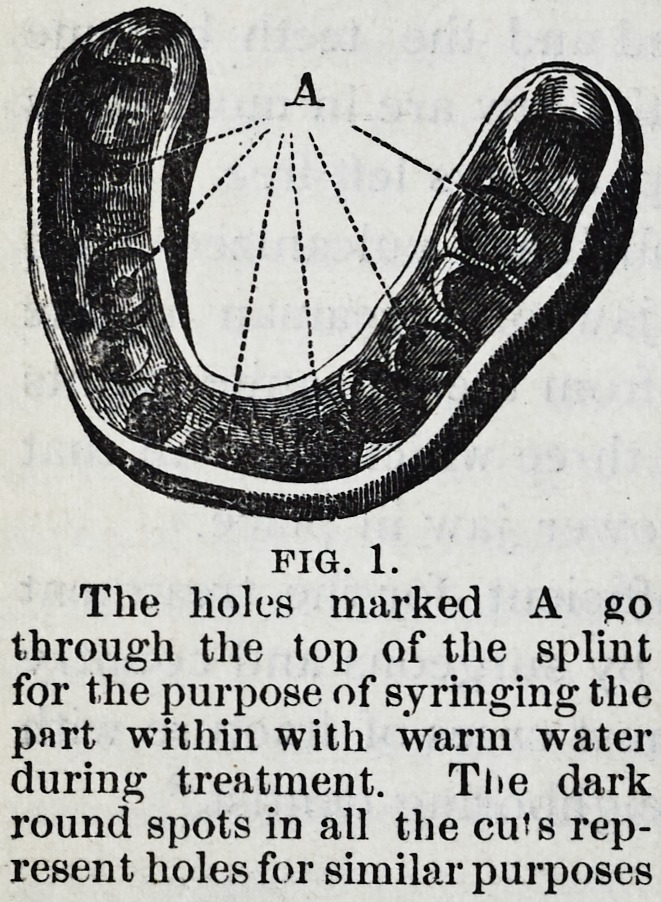


**FIG. 2. f2:**
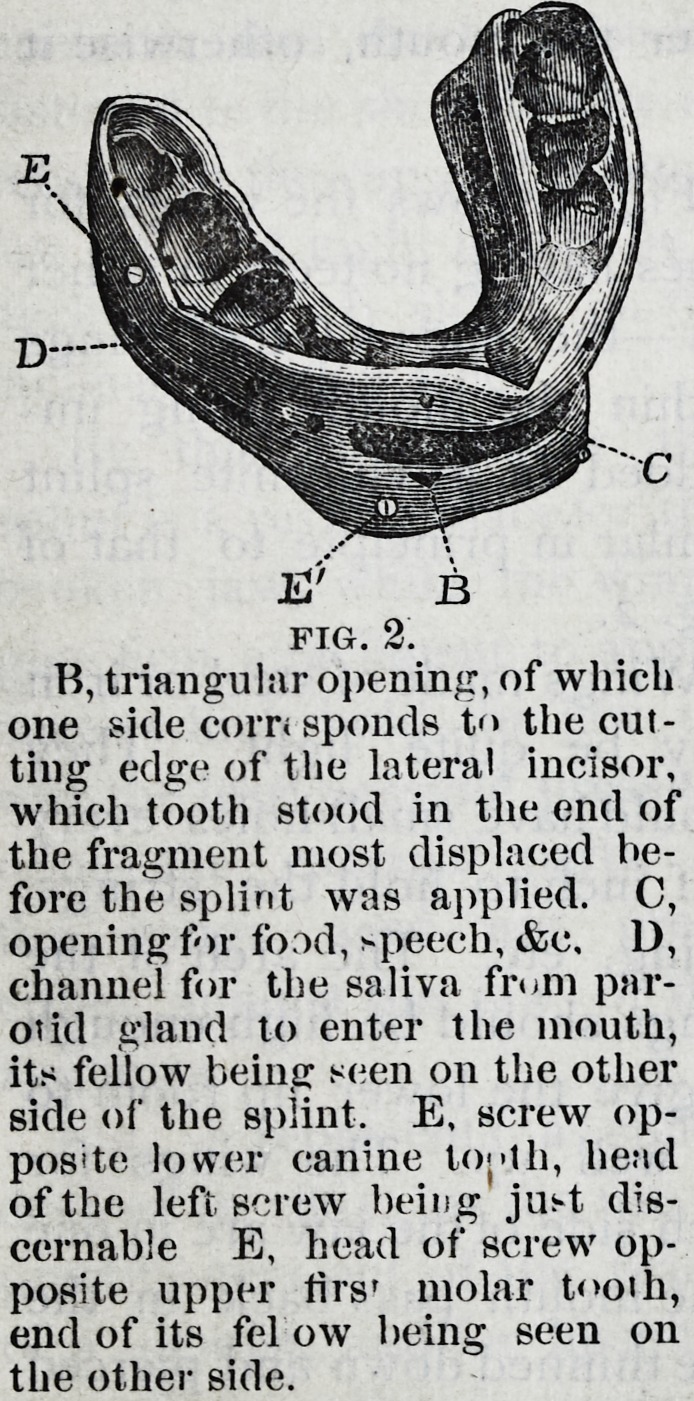


**FIG. 3. f3:**
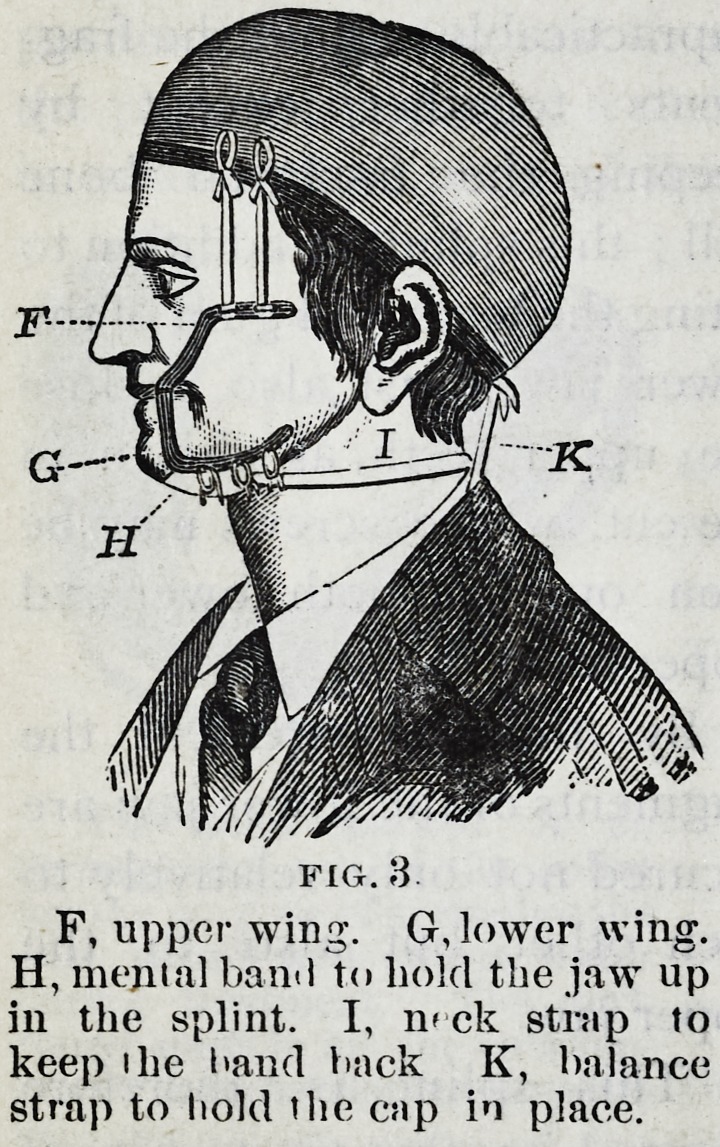


**FIG 4. f4:**
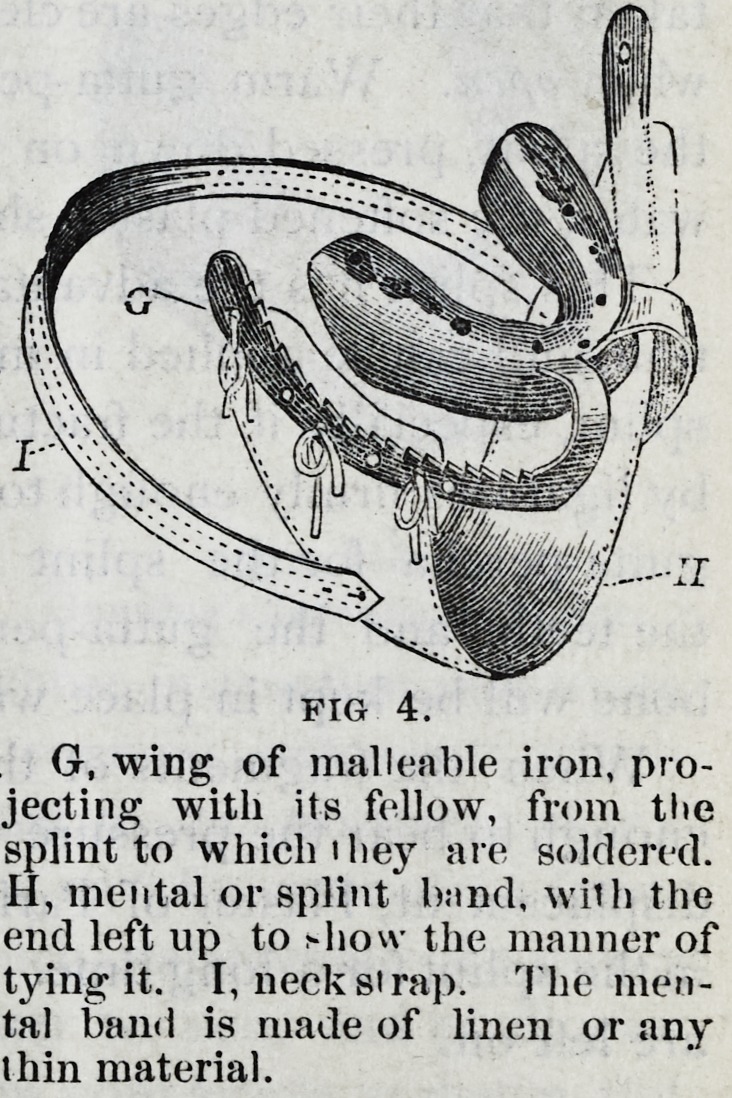


**FIG. 642. f5:**
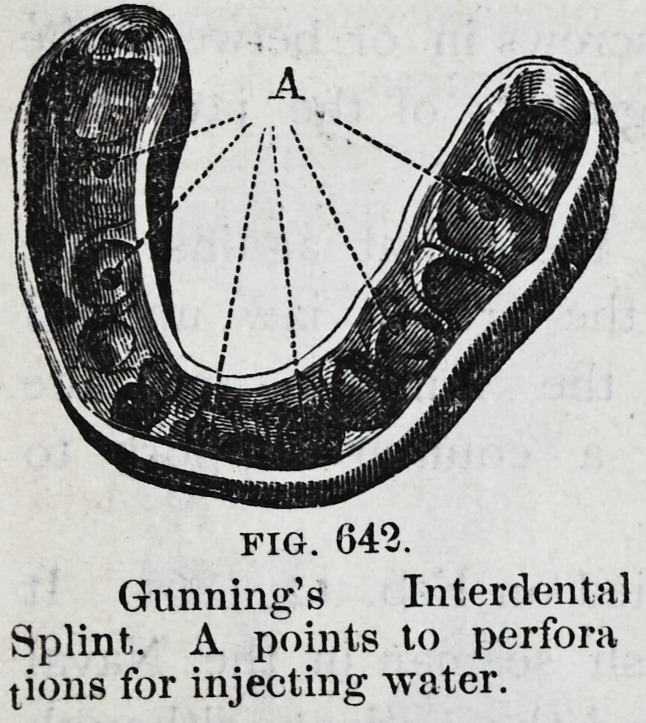


**FIG. 643. f6:**
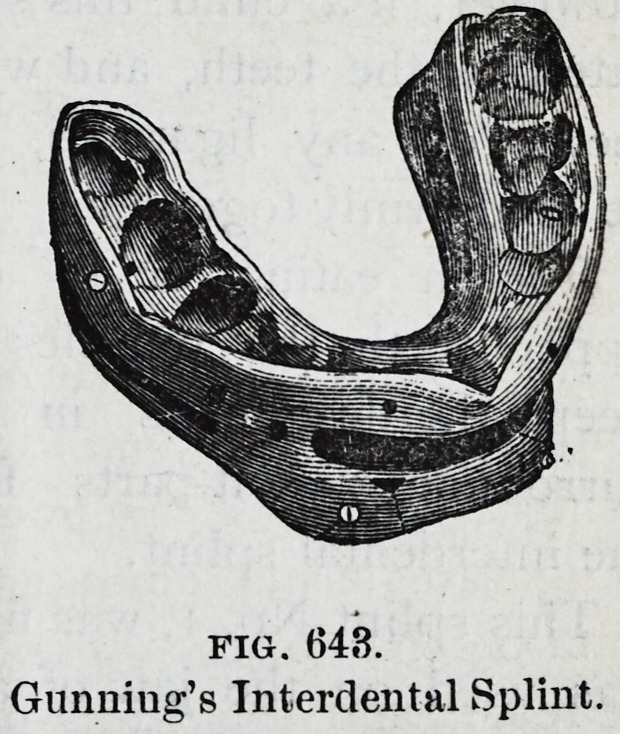


**FIG. 644. f7:**
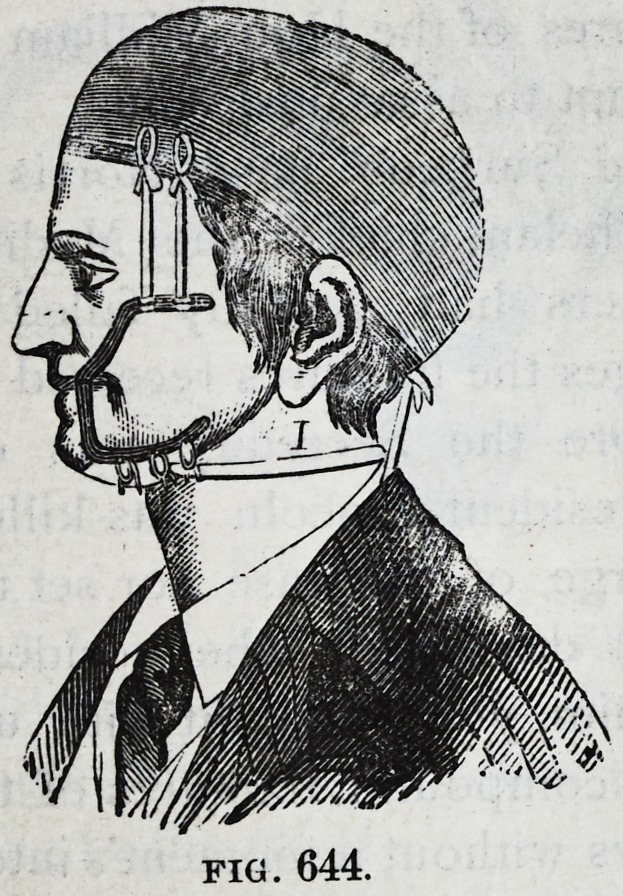


**Fig. 4. f8:**